# Supplementary material for: Oil Media on Paper: Investigating the Interaction of Cold-Pressed Linseed Oil with Paper Supports with FTIR Analysis
Source: Polymers (Basel). 2023 Jun 2;15(11):2567. doi: 10.3390/polym15112567 (PMC10255726; doi:10.3390/polym15112567)
Supplement: Supplementary file 1 [file polymers-15-02567-s001.zip › polymers-2299397-supplementary.pdf]

SUPPORTIVE INFORMATION

Tables

Table S1 Description of the materials used for the preparation of mock-ups, as provided by their manufacturers.

1.	Cold-pressed Linseed oil (CP)	An oil extracted without the use of heat, *Windsor and Newton*
2.	Refined linseed oil (RF)	A low-viscosity alkali refined oil of pale colour which is a slower drying variant of the linseed oils, *Windsor and Newton*
3.	Stand oil (StL)	A pale viscous oil that slows drying while imparting a tough, smooth enamel finish with no brush marks and excellent levelling, *Windsor and Newton*
4.	*Cotton (C)*	Cotton pHotonTM high purity paper by the Munktel paper Mill, *by Conservation by Design Limited, UK*
3.	*Montval (M)*	Montval watercolour paper, complying with ISO Standard 9706 requirements for permanence, by *Canson®* , France
4.	*Kraft (K)*	A wrapping paper*, by Dionysopoulos paper trade, Athens, Greece*

Table S2 Characteristic infrared absorption bands for cellulosic fibres [27, 33-39]*

Position (cm-1)	*Assignment*
3600-3000 (s, br)	*ν*(OH) free, hydroxyl stretching vibrations
3000-2800	*ν*(C-H), carbon - hydrogen stretching vibrations
1738	*υ*(C=O), ester
1650	δH-O-H bending (absorbed water)
1595	*υ*(C=C) aromatic in-plane
1457, 1369, 1337, 1248, 1236	exocyclic CH_2 deformations of the glucose units
1429 (m), 1316 (m-s)	δC-C-C + *δip*C-O-H, endocyclic C-C-H bends
1205	CO stretching
1110, 1054, 1031	combination of C-O stretch and C-O bend
896	C-C[1]-H deformation, marker of the β-glycosidic linkage
710, 663, 604, 557, 433	appear due to ring vibrational modes

Table S3 Characteristic infrared absorption bands for lignocellulosic papers [27, 37]

Position (cm^{-1})	*Assignment*
1738 (m-s)	*ν*C=O, esters in hemicellulose fraction
1652 (m-s)	νC=O (conjugated), coniferal aldehyde
1590	generally attributed to aromatics and possibly, to carboxylates
1505	aromatics, a lignin marker
1450	recorded in historic papers
~1265	broad absorbance due to C-O of guaiacyl ring of lignin residues
~900	glycosidic linkages in polysaccharide units
808	typical of hemicelluloses
1202–1204	mainly due to the exocyclic CH_2 twisting of the glucose rings with contributions from other vibrations, lignin removal marker

1050, 1030	various C–O vibrations of the polysaccharide structure, lignin removal marker

Table S4 Common vibrations in triacylglycerols [27]

Saturated	Unsaturated	
Position cm^{-1}	Position cm^{-1}	Assignment
	3450 (w,br)	
3450 (w,br)	3011 (w, br)	*ν*O-H, *hydroxyls, hydroperoxides*
	3011 (m-w)	*ν* =C-H (cis), *unconjugated cis double bonds*
2931	2958 (sh, m)	νasCH3, hydrocarbon chain
	2928 (s)	*ν*asC-H in CH2
2859	2875 sh, vw	*ν*sC-H in CH3
2828	2855 s	*ν*sC-H in CH2, symmetric stretching
1727 vs		*ν*C=O, *saturated ketones*
	1743 vs	*νC=O, ester carbonyl*
	1653 (w)	*ν*C=C, *conjugated trans double bonds*
1469 m-s	1464 m-s	*δ*CH2+ *δ*asCH3 bending vibrations
1390		*τ*CH2
1371 m	1379 m	*δ*sCH3
1334 w	1334 w	*w*CH2
1298 m-w	1298 m-w	
1278 m	1278 m	
1260 m	1260 m	
1242 m	1242 m	
1218 m	1218 m	
1196 m	1196 m	
1177 s	1169 s	*νa*C-O_C ester link
1110 m-w	1110 m-w	*νb*C-O-C ester link
1050, 1009, 982	1050	*ν*C-C
	977 w	*δ*oop +C-H (trans)
894		*ρ*CH3<br>*δ*oop =C-H (cis)
		*δ*oop =C-H (cis)
716		*ρ*CH2

Table S5 Neat linseed oil formulations at 0 days of ageing and after 40 days of air drying: integral calculations

Band	1820-1570				

	Mock-up #1	*Mock-up #2*	*Mock-up 3#*	*Average*	*STDV.p*
CP 0	69.33	68.76	68.91	69.00	0.24
CP 0-40	145.2	143.6	132.2	140.33	5.79
RF 0	70.17	69.78	69.46	69.80	0.29
RF 0-40	98.56	104.7	94.14	99.13	4.33
StL 0	59.89	59.68	59.69	59.75	0.10
StL 0-40	82.82	86.51	112.1	93.81	13.02
Band	1450-400				
	Mock-up #1	*Mock-up #2*	*Mock-up 3#*	*Average*	*STDV.p*
CP 0	70.14	60.56	76.58	69.09	6.58
CP 0-40	215.43	212.2	183.3	203.64	14.45
RF 0	83.03	91.72	103.2	92.65	8.26
RF 0-40	142.8	158.2	138.43	146.48	8.48
StL 0	15.38	36.36	29.45	27.06	8.73
StL 0-40	75.18	90.66	88.77	84.87	6.90
Integral ratio					
	CP 0-40/CP 0	RF 0-40/RF 0	StL 0-40/StL0		
1820-1570	2.03	1.42	1.57		
1450-400	2.95	1.58	3.14		

Table S6 Neat linseed oil formulations: Ratio of integrals (1900-1550 cm-1): (3200-2800 cm-1) [31]

	Mock-up#1	*Mock-up#2*	*Mock-ups#3*	*Averag*	*STDV.p*
CP 0	0.55	0.55	0.55	0.55	0.00
CP 0-40	1.19	1.15	1.05	1.13	0.06
CP 2	1.41	1.42	1.46	1.43	0.02
CP 4	1.41	1.38	1.38	1.39	0.02
CP 7	1.24	1.26	1.26	1.25	0.01
CP 14	1.19	1.35	1.33	1.29	0.07
CP 21	1.39	1.39	1.39	1.39	0.00
CP 28	1.39	1.39	1.37	1.38	0.01
RF 0	0.61	0.61	0.62	0.61	0.00
RF 0-40	0.85	0.89	0.79	0.84	0.04
RF 2	1.44	1.31	1.28	1.35	0.07
RF 4	1.34	1.33	1.21	1.29	0.06
RF 7	1.43	1.45	1.20	1.36	0.11
RF 14	1.26	1.24	1.23	1.24	0.01
RF 21	1.29	1.29	1.29	1.29	0.00
RF 28	1.24	1.30	1.32	1.29	0.04
StL 0	0.50	0.51	0.51	0.51	0.00
StL 0-40	0.70	0.71	0.87	0.76	0.08
StL 2	0.93	0.81	1.04	0.93	0.09
StL 4	1.00	0.90	0.91	0.94	0.05

StL 7	0.89	0.66	0.64	0.73	0.11
StL 14	0.55	0.55	0.55	0.55	0.10
StL 21	1.19	1.15	1.05	1.13	0.01
StL 28	1.41	1.42	1.46	1.43	0.00

Table S7 Neat linseed oil formulations: Ratio of derivatives' integrals (1730-1695 cm-1): (1760-1730 cm-1) [32]

	Mock-up#1	*Mock-up#2*	*Mock-up#3*	*Average*	*STDV.p*
CP 0	0.27	0.27	0.27	0.27	0.00
CP 0-40	0.21	0.17	0.14	0.18	0.03
CP 2	0.35	0.36	0.33	0.35	0.01
CP 4	0.38	0.42	0.42	0.41	0.02
CP 7	0.42	0.44	0.44	0.43	0.01
CP 14	0.77	0.83	0.83	0.81	0.03
CP 21	1.30	1.28	1.33	1.31	0.02
CP 28	1.79	1.79	1.80	1.79	0.00
RF 0	0.27	0.28	0.27	0.27	0.00
RF 0-40	0.13	0.13	0.13	0.13	0.00
RF 2	0.27	0.17	0.18	0.20	0.04
RF 4	0.39	0.39	0.31	0.37	0.04
RF 7	0.47	0.47	0.35	0.43	0.06
RF 14	0.79	0.79	0.80	0.79	0.00
RF 21	1.25	1.24	1.24	1.24	0.01
RF 28	1.73	1.68	2.18	1.86	0.23
StL 0	0.28	0.28	0.28	0.28	0.00
StL 0-40	0.22	0.22	0.22	0.22	0.00
StL 2	0.21	0.20	0.23	0.21	0.01
StL 4	0.30	0.27	0.28	0.28	0.01
StL 7	0.33	0.22	0.26	0.27	0.05
StL 14	0.27	0.27	0.27	0.27	0.07
StL 21	0.21	0.17	0.14	0.18	0.01
StL 28	0.35	0.36	0.33	0.35	0.00

Table S8 Oil-impregnated mock-ups: Ratio of derivatives integrals (1730-1695 cm-1): (1760-1730 cm-1)

	Mock-up#1	*Mock-up#2*	*Mock-up#3*	*Average*	*STDV.p*
CCP 0	0.76	0.67	0.77	0.73	0.05
CCP 2	0.87	0.89	0.89	0.88	0.01
CCP 4	0.98	0.98	0.98	0.98	0.00
CCP 7	1.29	1.29	1.26	1.28	0.01
CCP 14	1.53	1.44	1.28	1.41	0.10
CCP 21	2.01	1.92	1.93	1.96	0.04
CCP 28	2.06	1.94	1.71	1.90	0.14
MCP 0	0.56	0.58	0.50	0.55	0.03

MCP 2	0.52	0.45	0.49	0.49	0.03
MCP 4	0.49	0.34	0.50	0.44	0.08
MCP 7	0.61	0.71	0.57	0.63	0.06
MCP 14	0.54	0.71	0.56	0.61	0.08
MCP 21	0.84	0.71	0.72	0.75	0.06
MCP 28	0.68	0.67	0.67	0.67	0.01
KCP 0	0.57	0.56	0.60	0.58	0.02
KCP 2	0.46	0.39	0.32	0.39	0.06
KCP 4	0.55	0.49	0.42	0.49	0.05
KCP 7	0.61	0.41	0.34	0.46	0.12
KCP 14	0.81	0.63	0.87	0.77	0.10
KCP 21	0.91	0.92	0.71	0.85	0.10
KCP 28	1.07	0.99	1.05	1.04	0.03

(*) Note: Numbering of references respond to those listed in the paper.

Figures

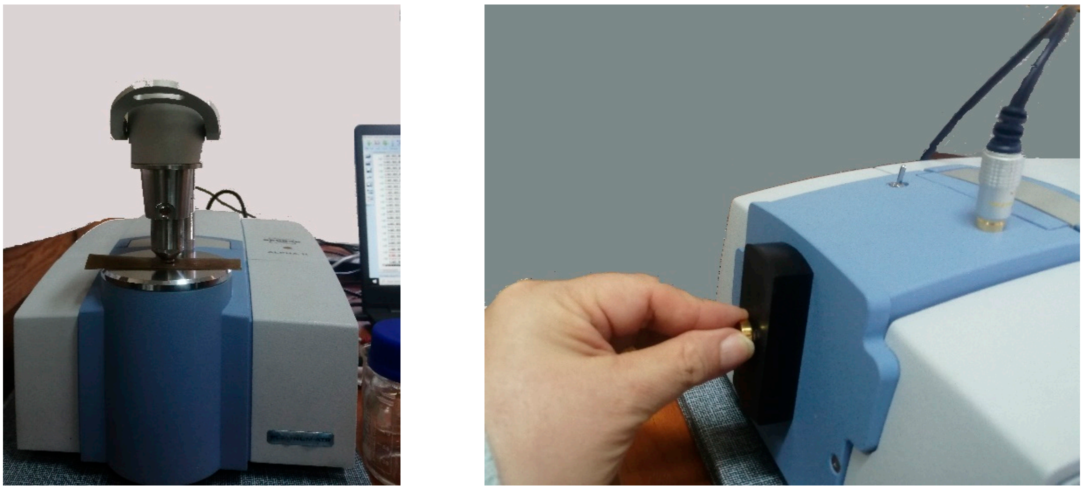

(a) (b)

Figure S1 Images of the FTIR methodology used for the different types of analysis: (a) the set-up for the ATR-FTIR analysis for plain paper and oil-impregnated mock-ups, and (b) the set-up for Reflectance FTIR for the oil films derived from the oil extraction of the oil-impregnated mock-ups

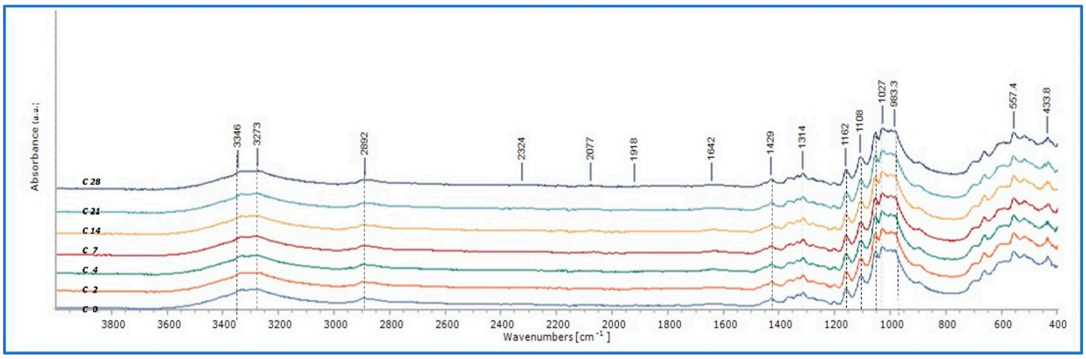

Figure S2 ATR-FTIR spectra of plain Cotton (C) mock-ups at all stages of artificial ageing (0-28 days), in an overlay display.

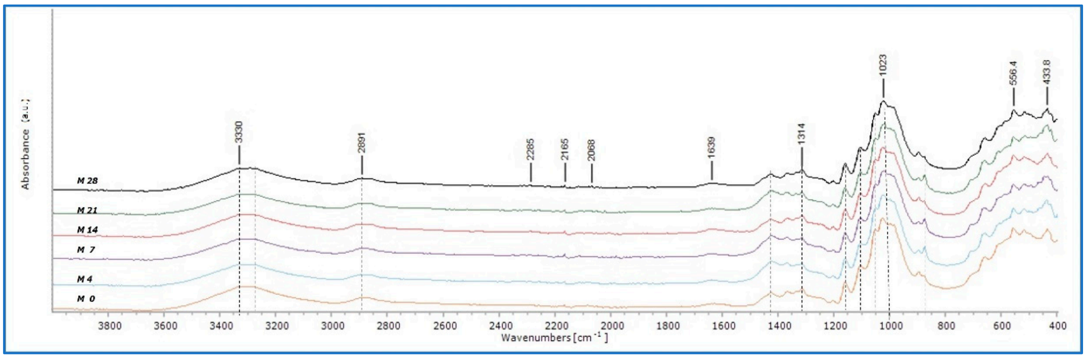

Figure S3 ATR-FTIR spectra of plain Montval (M) mock-ups at all stages of artificial ageing (0-28 days), in an overlay display.

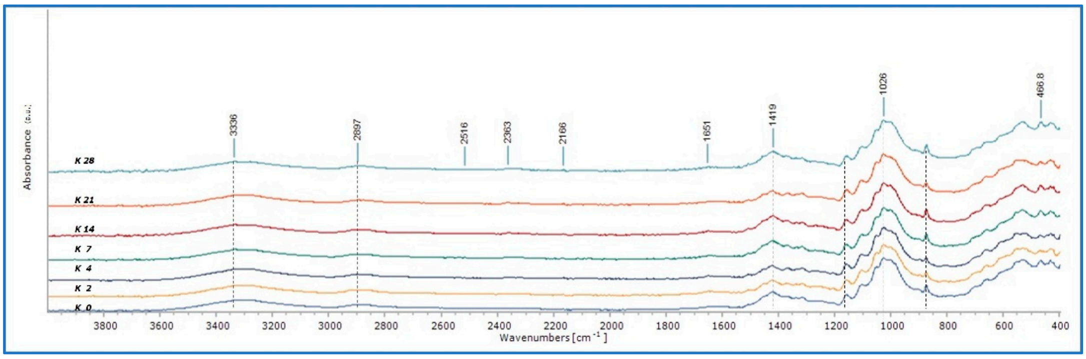

Figure S4 ATR-FTIR spectra of plain Kraft (K) mock-ups at all stages of artificial ageing (0-28 days), in an overlay display.

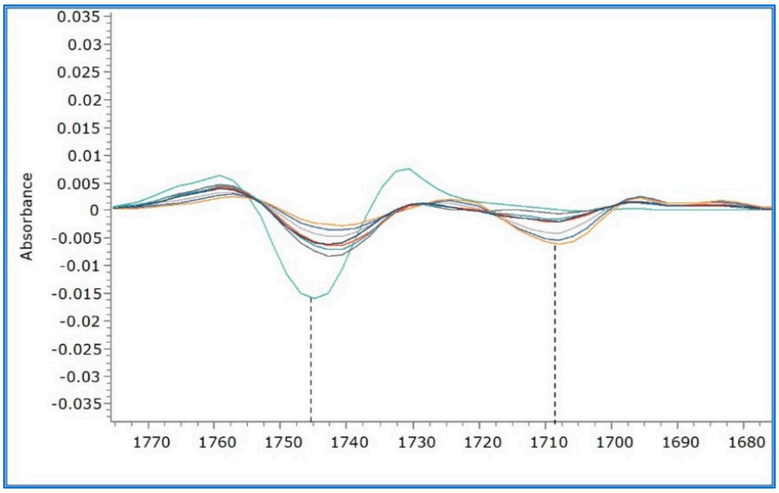

Figure S5 The derivatives of the CP spectra at all ageing stages, on the band 1760-1700 cm^{-1}

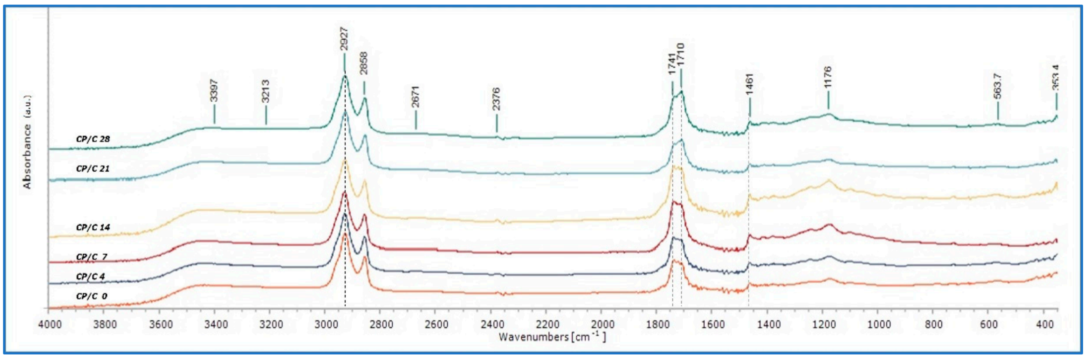

Figure S6 The reflection-FTIR spectra of CP extraction from Cotton oil-impregnated mock-ups upon ageing.

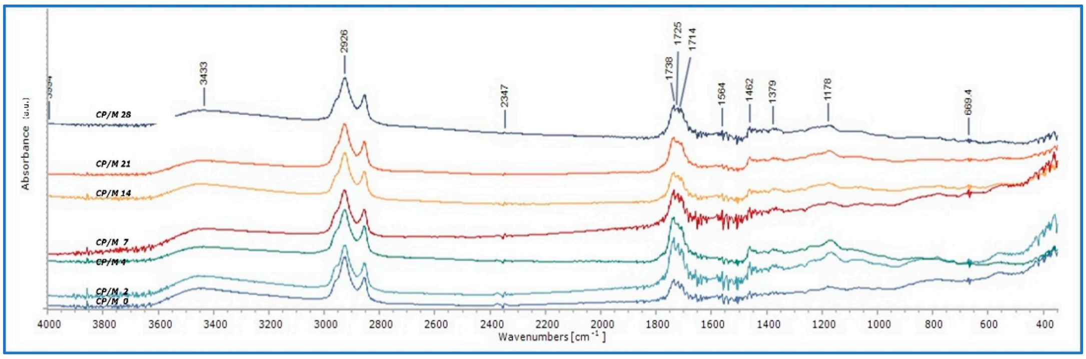

Figure S7 The reflection-FTIR spectra of CP extraction from Montval oil-impregnated mock-ups upon ageing.

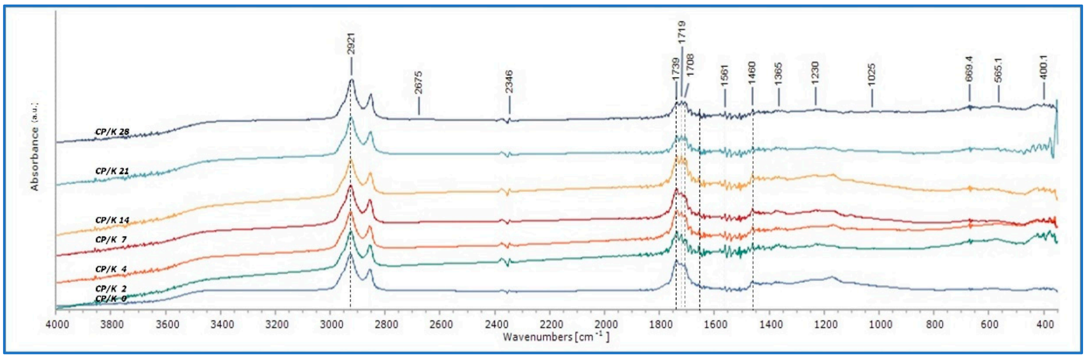

Figure S8 The reflection-FTIR spectra of CP extraction from Kraft oil- impregnated mock-ups upon ageing.

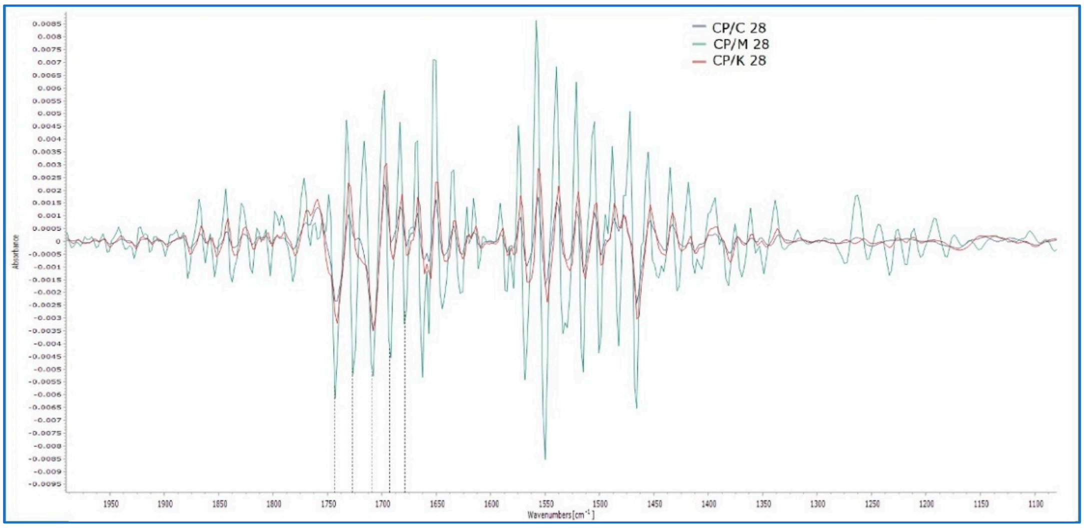

Figure S9 Detail image of the derivatives of CPs reflection spectra that derived from the extraction of C, M, and K oil-impregnated mock-ups, at the final stage of ageing. The formation of several peaks on the band 1700-1550cm-1 is clear.
